# COVID-19 Hyperinflammation: What about Neutrophils?

**DOI:** 10.1128/mSphere.00367-20

**Published:** 2020-06-24

**Authors:** Athanasios Didangelos

**Affiliations:** a Mayer IgA Nephropathy Laboratory, University of Leicester, Leicester, United Kingdom; National Institute of Allergy and Infectious Diseases

**Keywords:** COVID-19, SARS-CoV-2, coronavirus, inflammation, neutrophil

## Abstract

COVID-19 is often related to hyperinflammation that drives lung or multiorgan injury. The immunopathological mechanisms that cause excessive inflammation are under investigation and constantly updated. Here, a gene network approach was used on recently published data sets to identify possible COVID-19 inflammatory mechanisms and bioactive genes. First, network analysis of putative SARS-CoV-2 cellular receptors led to the mining of a neutrophil-response signature and relevant inflammatory genes. Second, analysis of RNA-seq data sets of lung cells infected with SARS-CoV-2 revealed that infected cells expressed neutrophil-attracting chemokines. Third, analysis of RNA-seq data sets of bronchoalveolar lavage fluid cells from COVID-19 patients identified upregulation of neutrophil genes and chemokines. Different inflammatory genes mined here, including TNFR, IL-8, CXCR1, CXCR2, ADAM10, GPR84, MME, ANPEP, and LAP3, might be druggable targets in efforts to limit SARS-CoV-2 inflammation in severe clinical cases. The possible role of neutrophils in COVID-19 inflammation needs to be studied further.

## Abstract (precis)

COVID-19 is often related to hyperinflammation that drives lung or multiorgan injury. The immunopathological mechanisms that cause excessive inflammation are under investigation and constantly updated. Here, a gene network approach was used on recently published data sets to identify possible COVID-19 inflammatory mechanisms and bioactive genes. First, network analysis of putative SARS-CoV-2 cellular receptors led to the mining of a neutrophil-response signature and relevant inflammatory genes. Second, analysis of RNA-seq data sets of lung cells infected with SARS-CoV-2 revealed that infected cells expressed neutrophil-attracting chemokines.

## OPINION/HYPOTHESIS

While in most individuals with SARS-CoV-2 pneumonia host immune response clears the lung infection, in many severe cases an aggressive and uncontrolled inflammatory response exacerbates viral lung damage and can contribute to respiratory failure. The epidemiology and pathophysiology of COVID-19 are constantly being updated, but it is becoming apparent that disease severity is related not only to alveolar epithelial cell damage caused by SARS-CoV-2 but also to hyperinflammation that can drive lung and multiorgan injury and mortality via cytokine storm and sepsis (1). Although different immunosuppressive and immunoregulatory options are being considered, including corticosteroids (1, 2), other studies recommend caution with immunosuppression given that regulated inflammation is necessary for an effective antiviral response (3). More ideas and studies are needed to dissect effective versus damaging host inflammatory responses in COVID-19 and to understand the mechanisms of hyperinflammation in severe cases. The role and function of infiltrating and peripheral myeloid cells in COVID-19 lung injury, cytokine storm, and fatal sepsis (4) are also not well characterized, and efforts to understand the complex immunological landscape are evolving. This paper utilized publicly available network and pathway analysis tools on recently published COVID-19 data sets to explore possible inflammatory signatures linked to SARS-CoV-2 infection in humans.

**FIG 1:**
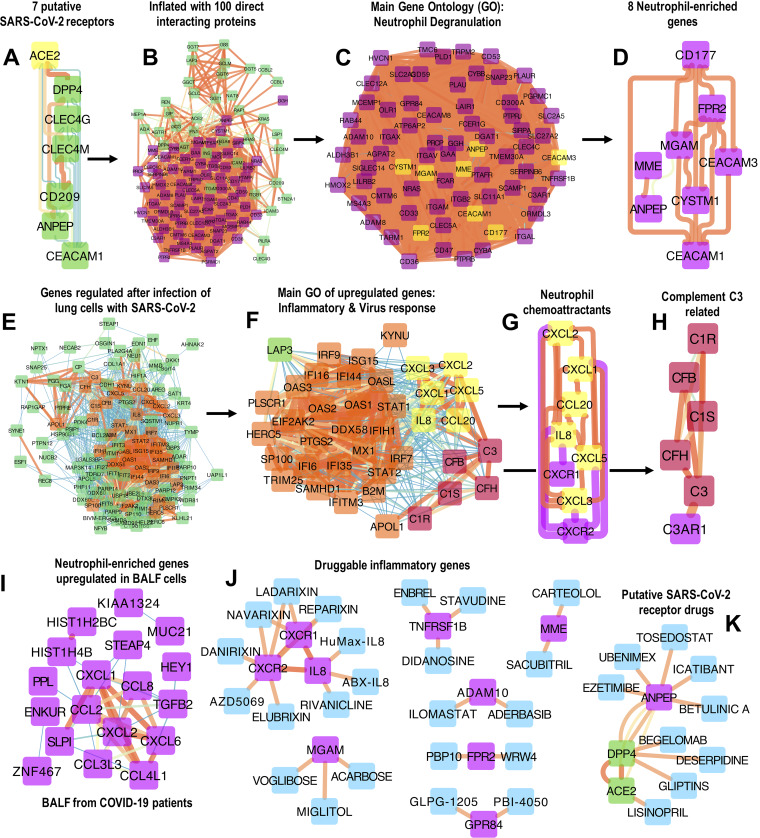
(A) ACE2 and 6 related putative SARS-CoV-2 receptors on epithelial cells. ACE2, DPP4, and ANPEP are peptidases. (B) The 7 putative SARS-CoV-2 receptors were inflated in StringDB by adding up to 100 directly interacting proteins with direct association to the 7 input proteins using 1st shell interactions and StringDB default settings. (C and D) The main gene ontology of this inflated network is “Neutrophil Degranulation” (C) (http://amigo.geneontology.org/amigo/term/GO:0043312) and includes 8 neutrophil-enriched genes isolated in panel D. The gene ontology of these proteins was examined in the Amigo database. (E) One hundred thirteen genes were differentially regulated in RNA-seq data sets of human alveolar adenocarcinoma (A549) and human bronchial epithelial cells infected with SARS-CoV-2 *in vitro* (study details, data sets, and statistical analysis can be found in reference 14). (F) Following virus infection, most significantly upregulated genes were related to inflammatory and interferon responses. (G and H) Six classic neutrophil chemokines were upregulated in these cells following infection with SARS-CoV-2, depicted in panel G, as well as C3 and related complement pathway genes depicted in panel H. (I) Eighteen neutrophil-enriched and neutrophil chemotaxis genes were upregulated in an RNA-seq data set of BALF cells collected from 2 COVID-19 patients versus 3 healthy BALF donors (study details, data sets, and statistical analysis can be found in reference 16). (J and K) Putative druggable targets with likely neutrophil proinflammatory function derived from the analysis. Approved and experimental drugs with validated pharmacological evidence are presented as interaction networks. Protein-drug interactions were retrieved from DGIdb (v3.02; http://www.dgidb.org/search_interactions) and were curated (DrugBank [https://www.drugbank.ca/]) to exclude nonvalidated and false-positive interactions. All protein-protein interaction networks were developed in Cytoscape with cumulative protein-protein interaction scores computed in StringDB (v11) (https://string-db.org/) using default interaction sources (experimental evidence, coexpression, gene fusion, cooccurrence, curated databases, and references in scientific literature text-mining).

A recent study highlighted 7 putative SARS-CoV-2 receptors including the now well-known ACE2 (5) together with peptidases DPP4 and ANPEP as well as pathogen-binding proteins CD209, CLEC4G, CLEC4M, and CEACAM1 (Fig. 1A). These proteins are expressed in different cell types and are implicated in the binding of coronaviruses on epithelial cells (6). To examine these proteins further, the small signature (Fig. 1A) was inflated in StringDB (https://string-db.org/) to construct a protein-protein interaction network with up to 100 proteins that directly interact with the 7 input proteins via a single path, using 1st shell interacting proteins and default interaction settings (Fig. 1B). Network inflation was performed to allow mining of possible functional pathways related to the 7 input proteins. Surprisingly, the main ontology of the expanded network (Fig. 1B) was “Neutrophil Degranulation” (gene ontology [GO] was examined in the Amigo database [http://amigo.geneontology.org/amigo/term/GO:0043312]) with 70 proteins in this category (Fig. 1C). There were also 8 neutrophil-enriched genes (neutrophil gene expression enrichment is available via the Human Protein Atlas, https://www.proteinatlas.org/search/blood_cell_category_rna%3Aneutrophil%3BCell+type+enriched+AND+sort_by%3Atissue+specific+score) including ANPEP, MME, MGAM, CD177, CEACAM1/3, FPR2 and CYSTM1 (Fig. 1D). Thus, coronavirus binding proteins (and directly associated proteins) might be involved in neutrophil or other related classic inflammatory mechanisms. Two of the neutrophil-specific genes, ANPEP and CEACAM1 (Fig. 1D), are coronavirus receptors (Fig. 1A), indicating that neutrophils could be infected by SARS-CoV-2, as they are by influenza virus (7).

The role of neutrophils in viral infections of the upper respiratory tract and their possible importance in therapeutic strategies is not entirely clarified (8). They are involved in early antiviral defense (9), but through degranulation and lysis, they can be cytotoxic during severe pneumonia, including from coronaviruses (10), and can also aggravate lung inflammation caused by influenza virus (11, 12). Neutrophil hyperinflammation is also likely in other severe viral infections such as hepatitis (8). In current COVID-19 literature, an increased peripheral neutrophil-to-lymphocyte ratio is observed in severe cases and is likely associated with unfavorable prognosis (13). The mechanisms behind this are not understood, and not much is known regarding neutrophil activity in SARS-CoV-2-infected lungs. COVID-19 lung damage in some patients might involve dysregulated neutrophil activity.

To examine possible neutrophil involvement following SARS-CoV-2 infection, a published RNA-seq data set of human alveolar adenocarcinoma (A549) cells infected with SARS-CoV-2 *in vitro* (14) was analyzed. Experimental and statistical details related to this data set are described in reference 14. Differentially regulated genes are visualized as a protein-protein interaction network developed using default settings in StringDB (Fig. 1E). The signature of SARS-CoV-2-infected lung cells contains 39 inflammation and viral-response genes, including classic inflammatory mediators and interferon pathway genes (Fig. 1F). Notably, infected lung cells overexpressed 6 chemokines that belong to the human ontology “Neutrophil Chemotaxis” (http://amigo.geneontology.org/amigo/term/GO:0030593; Amigo gene ontology) and include the classic neutrophil chemoattractants CXCL1, CXCL2, CXCL3, CXCL5, IL-8 (CXCL8), and CCL20 (Fig. 1G), suggesting that these cells can express neutrophil chemokines after SARS-CoV-2 infection. The receptors for these chemokines (CXCR2 and CXCR1; IL-8 receptor) are neutrophil-enriched genes like CXCL1, CXCL5, IL-8, ANPEP, and CEACAM1 (https://www.proteinatlas.org/search/blood_cell_category_rna%3Aneutrophil%3BCell+type+enriched+AND+sort_by%3Atissue+specific+score; Human Protein Atlas). SARS-CoV-2-infected lung cells also overexpressed complement C3 and associated pathway activation genes (Fig. 1H), including the receptor for the C3a anaphylatoxin (C3AR1). C3 and complement activation have been recently involved in acute respiratory distress syndrome (ARDS) with systemic inflammation and lung neutrophilia (15).

Finally, analysis of a published RNA-seq data set of human bronchoalveolar lavage fluid (BALF) cells from 2 hospitalized COVID-19 patients (16) revealed that 18 (Fig. 1I) neutrophil-enriched genes (PPL, ENCUR, STEAP4, SLP1, MUC21, HEY1, MUC21, and CXCL1) and neutrophil chemotaxis genes (CXCX2, CXCL6, CCL8, CCL2, TGFB2, CCL3L3, and CCL4L4) were upregulated in COVID-19 BALF cells, further supporting likely involvement of neutrophils in COVID-19 lungs. Experimental and statistical details related to this data set are described in reference 16. Caution is needed in assessing the statistical robustness of these data given that BALF from only 2 COVID-19 patients was sequenced in this study, and there was no information on the severity or clinical outcome of these subjects.

Are neutrophils and related inflammatory mechanisms likely targets in COVID-19 complications? This is a difficult question given the complexity of innate immune responses and the importance of neutrophils in early antiviral defense as well as their role in secondary bacterial and fungal infections that are common comorbidities in COVID-19 patients (17). Universal suppression of neutrophils or other myeloid cell types is not trivial, and the clinical evidence on the use of steroids in COVID-19 is inconclusive. Nevertheless, it might be possible to target specific inflammatory mechanisms to prevent lung injury or systemic hyperinflammation and cytokine storm. The target mining presented here includes multiple druggable proteins (Fig. 1J and K) such as neutrophil-attracting chemokine signaling (Fig. 1J), and neutrophil-relevant inflammatory entities (Fig. 1J), as well as SARS-CoV-2 receptors (Fig. 1K). Approved and experimental drug-protein interactions were retrieved from the DGIdb drug-protein interaction database (http://www.dgidb.org/search_interactions) using default filters.

IL-8 (Fig. 1J), a cardinal neutrophil chemoattractant and product of activated neutrophils, can be blocked by neutralizing antibodies (HuMax-IL8). The neutrophil chemokine receptors CXCR1 and CXCR2 are also targeted by experimental drugs, including the CXCR2 blocker AZD5069 (Fig. 1J). Other interesting inflammatory proteins include the monocyte and neutrophil-expressed genes TNF-receptor-2 (TNFR2 or TNFRSF1B), GPR84, and ADAM10 (Fig. 1J). TNFR2 is clinically blocked by the receptor-antagonist etanercept (Enbrel) while TNF is targeted by neutralizing antibodies (infliximab). TNF has a well-established role in neutrophil activation and prolongs neutrophil survival (18). GPR84 and ADAM10 are targeted with experimental drugs including the trialed GPR84 blocker GLPG-1205 and ADAM10 inhibitor ilomastat (Fig. 1J). FPR2 (formyl peptide receptor), MME (neprilysin), and MGAM (maltase-glucoamylase) (Fig. 1J) are directly involved in neutrophil recruitment and activity. ANPEP (aminopeptidase-N), a likely coronavirus receptor (Fig. 1A) and neutrophil-specific gene (Fig. 1D), is potentially blocked by approved drugs ezetimibe and icatibant, while there is experimental evidence that it interacts with tosedostat and ubenimex. It is, however, unlikely that binding of these drugs to the aminopeptidase will interfere with viral binding. Ubenimex is also an inhibitor of LAP3 (leucine aminopeptidase-3), the only gene shared between the coronavirus receptor inflated network (Fig. 1B) and SARS-CoV-2-infected lung cells (Fig. 1F).

Neutrophil recruitment and related activity might exacerbate COVID-19 immunopathology. In contrast to universal immunosuppression, specific inflammatory pathways could be targeted in COVID-19 hyperinflammation. These approaches can be also combined with antivirals. For instance, oseltamivir (Tamiflu) was recently shown to reduce neutrophil lung recruitment when combined with CXCR2 antagonists in influenza virus-infected mice (19). Moreover, it is perhaps worth noting that some currently investigated COVID-19 therapeutic options include hydroxychloroquine, azithromycin, and colchicine. All three have known antineutrophil effects. Finally, it is important to highlight that this paper utilized publicly available bioinformatics tools to analyze a limited number of high-throughput gene expression data sets from *in vitro* and *in vivo* SARS-CoV-2 infections. The likely involvement of neutrophil signatures and related inflammatory pathways presented here is unbiased and should be used with caution to generate hypotheses for future experimental and translational studies. Interestingly, after this manuscript was submitted for consideration, the likely pathological role of lung neutrophil activity (formation of extracellular traps) in COVID-19 clinical complications was published (20). More experimental and clinical work is needed to validate these findings and to inform possible anti-inflammatory interventions for severe cases. The immunopathological mechanisms driving COVID-19 are unclear, are continuously updated, and will evolve drastically in the future.
